# Metabolisable energy content in canine and feline foods is best predicted by the NRC2006 equation

**DOI:** 10.1371/journal.pone.0223099

**Published:** 2019-09-27

**Authors:** Juliane Calvez, Mickael Weber, Claude Ecochard, Louise Kleim, John Flanagan, Vincent Biourge, Alexander J. German

**Affiliations:** 1 Royal Canin Research Center, Aimargues, France; 2 Institute of Ageing and Chronic Disease, University of Liverpool, Neston, Cheshire, United Kingdom; 3 Institute of Veterinary Science, University of Liverpool, Neston, Cheshire, United Kingdom; University of Illinois, UNITED STATES

## Abstract

Although animal trials are the most accurate approach to determine the metabolisable energy (ME) content of pet food, these are expensive and labour-intensive. Instead, various equations have been proposed to predict ME content, but no single method is universally recommended. Data from canine and feline feeding studies, conducted according to Association of American Feed Control Officials recommendations, over a 6-year period at a single research site, were utilised to determine the performance of different predictive equations. Predictive equations tested included the modified Atwater (MA equation), NRC 2006 equations using both crude fibre (NRC 2006_cf_) and total dietary fibre (NRC 2006_tdf_), and new equations reported in the most recent study assessing ME predictive equations (Hall equations; PLoS ONE 8(1): e54405). Where appropriate, equations were tested using both predicted gross energy (GE) and GE measured by bomb calorimetry. Associations between measured and predicted ME were compared with Deming regression, whilst agreement was assessed with Bland-Altman plots. 335 feeding trials were included, comprising 207 canine (182 dry food; 25 wet food) and 128 feline trials (104 dry food, 24 wet food). Predicted ME was positively associated with measured ME whatever the equation used (*P*<0.001 for all). Agreement between predicted and actual ME was worst for the MA equation, for all food types, with evidence of both a systematic bias and proportional errors evident for all food types. The NRC 2006_cf_ and Hall equations were intermediate in performance, whilst the NRC 2006_tdf_ equations performed best especially when using measured rather than predicted GE, with the narrowest 95% limits of agreement, minimal bias and proportional error. In conclusion, when predicting ME content of pet food, veterinarians, nutritionists, pet food manufacturers and regulatory bodies are strongly advised to use the NRC 2006_tdf_ equations and using measured rather than predicted GE.

## Introduction

Most pet dogs and cats are fed complete and balanced diets, whereby all essential nutrients in the food are balanced relative to energy density [1]. With such diets, provided that sufficient food is consumed to meet daily energy requirements, the requirements for each nutrient will also be met. To determine the daily food requirements of an individual dog or cat, both the animal’s maintenance energy requirement and the energy density of the food must be known, with daily food intake determined from dividing the former by the latter [1]. The energy content of food is typically expressed using terms such as gross energy (GE), digestible energy (DE), and metabolisable energy (ME). The GE of a food is usually measured by completely combusting the food in a bomb calorimeter [2], whilst DE (the amount of energy available to the animal after digestion) is measured by subtracting the energy lost in faeces from the GE of the food [2]. The ME of a food is the energy potentially available for metabolism and is derived by subtracting energy lost in both urine and faeces from the GE of the food [2].

A food’s ME content is most accurately determined in an animal feeding trial, and protocols for such animal trials have been developed by the Association of American Feed Control Officials (AAFCO) [3]. However, since such trials are expensive and labour-intensive, the alternative is to predict ME content either with factorial equations, with the factors for each nutrient reflecting the heat of combustion and digestibility of the respective nutrients, or interactive methods, which base assumptions on the nutrients associated with energy digestibility [4]. The most commonly-used factorial equations have a 3-component design, whereby ME is determined by multiplying the content of crude protein, fat and nitrogen-free extract (NFE, which represents the carbohydrate fraction) by fixed energy values [5–7]. Atwater described the original method [5], which assumed a digestibility of 90, 97 and 96% for protein, fat and carbohydrate, respectively. However, given that the typical digestibility of most pet foods is between 75% and 83% [6], the original factors were subsequently modified for both dogs and cats [7,8], creating the so-called “modified Atwater” (MA) factorial equation (3.5 kcal/g of protein, 8.5 kcal/g of fat, 3.5 Kcal/g of NFE) that the 1985 National Research Council (NRC) report endorsed [9], and which AAFCO still recommends [3]. Whilst such an equation is simple to use, limitations include the fact in that it does not take account of species differences, differences in the actual digestibility of the food, or differences in fibre content [10]. As a result, this equation tends to underestimate energy content of highly-digestible foods whilst, at the same time, overestimating the energy content of foods of a lesser digestibility [11]. For this reason, others have recommended methods based on GE [6,7], fat (F) [6], crude protein (CP) [12], a method using near infrared reflectance spectroscopy [12] and the fibre content [9,13]. These latter studies prompted the NRC in 2006 to recommend equations that are based upon either crude fibre (CF) or total dietary fibre (TDF) [2,14], methods since adopted by FEDIAF, the European Pet Food Federation, in their guidelines [15].

Hall *et al*. (2013) conducted the most recent study of ME prediction in pet food, by examining over 500 cat and dog feeding studies and comparing the MA factorial equation with interactive methods based on CF [16]. Performance of both methods was equivalent, but neither performed as well as new equations based on their study data utilising GE, fat content, protein content, moisture content, and crude fibre content [16]. However, one methodological issue was the fact that the same data were used both to generate the predictive equation and then test its validity and, therefore, it is not surprising that the new equations better fitted the data [17,18]. Instead, confirming superiority of these alternative equations ideally requires cross-validation with an independent dataset [17,18]. A second methodological issue with the study was the fact that both dry and wet foods were included within the same linear regression analyses. This meant that the regressions effectively assessed two distinct groups within the same analysis, one (comprising wet foods) with low ME content and the other (comprising dry foods) with high ME content. In addition to energy content, wet and dry foods can differ in macronutrient content, with wet foods typically containing more protein and fat, but less carbohydrate and fibre that dry food. Since different predictive equations might perform differently for different food types (wet and dry), analysing data from the types separately is preferable. A final methodological issue was the fact that this, and many other previous studies, have used general linear regression when comparing measured ME with predicted ME from equations. However, since such experiments are actually method comparison studies, other statistical methods are arguably more appropriate. For example, the Clinical and Laboratory Standards Institute [19] recommends the use of Deming regression [20] and Bland-Altman plots [21] for assessing association and agreement, respectively, in method-comparison studies. These methods are also recommended for method-comparison studies in veterinary science [22]. Therefore, the purpose of the current study was to assess different equations for predicting ME content of cat and dog food using data from feeding trials conducted over a period of 5 years at a single research centre. We also chose to examine separately, the associations between dry and wet food in order to avoid issues of energy density affecting associations, and used statistical procedures recommended for method-comparison studies.

## Materials and methods

### Eligibility criteria for feeding trials

All feeding trials, conducted at a single location (Royal Canin Research Center, Aimargues, France), between January 2006 and December 2011 were reviewed. For inclusion, trials had to have been conducted according to according to AAFCO recommendations for Dog and Cat Food Metabolizable Energy Protocols (Method 1: Quantitative Collection) [3], meaning that details of proximate analysis and gross energy (GE) for both the diet and the faeces, as well as digestibility data from 6 animals were available (e.g. where GE had both been measured and predicted according to NRC 2006 equations [2]). Details of the diets included are summarised in Table 1, and details of all diets is given (S1 File).

**Table 1:** Composition of canine foods assessed in the study.

	Dry Food^1^ (n = 182)				Wet food^1^ (n = 25)			
	Mean	SD	Median	Range	Mean	SD	Median	Range
Measured GE^2^	4750	198.1	4570	4132–5458	1296	260.8	1240	740–1980
Predicted GE^2^	4817	204.8	4817	4199–5505	1284	292.9	1231	721–2101
Crude protein (%)	26.8	4.18	26.9	10.0–35.3	7.9	1.97	7.8	5.4–14.3
Crude fat (%)	16.0	3.27	16.0	8.2–28.7	4.7	2.08	4.5	1.6–9.5
Total dietary fibre (%)	8.6	4.60	7.1	2.1–27.7	2.4	0.83	2.6	1.1–3.9
Crude fibre (%)	3.3	3.04	2.4	0.9–17.5	1.7	0.31	1.5	1.5–2.5
Ash (%)	6.1	1.08	6.2	2.9–9.6	1.5	0.30	1.5	1.1–2.3
Moisture (%)	7.4	1.33	7.6	3.6–11.2	76.3	5.49	76.5	61.8–86.0

^1^ Values reported are on an as fed basis.

^2^GE: gross energy content of food in Kcal per kg, determined either by measurement [3] or prediction [2].

### Animals

In total, 77 dogs and 41 cats participated in the digestibility trials, with groups of 6 dogs or cats being selected for each trial on a rotational basis. All dogs were female neutered, their median age was 9y (mean ± standard deviation [SD] 8 ±3y; range 1-15y), from various breeds including: Beauceron, Bichon Frise, Boxer, Brittany, Cairn Terrier, Cavalier King Charles Spaniel, English Cocker Spaniel, English Setter, German Shepherd dog, Golden Retriever, Labrador Retriever, Miniature Schnauzer, Shetland Sheepdog, Springer Spaniel, White Swiss Shepherd dog, West Highland White Terrier, and Wire-Coated Dachshund. Cats were either male neutered or female neutered, with a median age of 9y (mean 9 ±1y, range 3-16y), and one of three breeds (domestic shorthair, Chartreux, and Japanese bobtail).

### Housing and husbandry

All dogs and cats used in the feeding trials were maintained in the colony of a commercial pet food company and were not client-owned. Regular health checks were conducted to ensure that the dogs and cats participating in the feeding trials remained healthy. Assessments performed included physical examination and clinicopathological assessments (e.g. blood chemistries and complete blood counts), which were conducted on a monthly and annual basis, respectively. In addition, the wellbeing of all animals was assessed daily by the caregivers who visually assessed signs of physical and mental health. Animals were only selected for trials if they were deemed to be healthy. No adverse events were reported during any of the food trials, none of the animals had to be withdrawn and no modifications to any of the experimental protocols were required.

Dogs were housed individually in indoor-outdoor runs (indoor 1.8–4.3 m²; outdoor 3.1–9.0 m², depending on dog size). Inside, temperature varied between 18–24°C, depending on season, and artificial light was provided between 07:30 and 17:00 if natural light was judged to be insufficient by animal handlers. Humidity within the kennels was not recorded. During the 9-day adaptation period, dogs were also walked for 1 hour per day, and had 3 hours per days in groups in outdoor pens (155–226 m², depending on dog size; 5 dogs per pen). During digestibility period (5 days), dogs were housed in their runs only, in order to prevent them from eating grass.

Cats were housed in closed indoor-outdoor runs. The size of all runs was 27 m², and there was a maximum of 7 cats per run, and the cat groups remained the same throughout the study. The runs with outdoor access were divided into an indoor part (of 13 m²) and an outdoor part (of 14 m²). Inside, temperature varied between 18–24°C, depending on season, and artificial light was provided between 07:30 and 17:00 if natural light was judged to be insufficient by animal handlers. Again, humidity data were not recorded. For all cats, care-givers stimulated play behaviour for approximately 2h, per run, per day. During digestibility period (5 days), cats were housed in individual lodges (1.4 m²) in order to obtain individual data. These lodges were environmentally enriched with raised platforms at different heights, to make best use of vertical space. All cats remained healthy for the duration of the studies.

### Feeding trial protocol, proximate analysis and measurement of the energy content of food

Trial protocols complied with European Union guidelines on animal welfare and were approved by the Royal Canin Committee for Animal Ethics and Welfare. Trials were conducted according to AAFCO Dog and Cat Food Metabolizable Energy Protocols (Method 1: Quantitative Collection) [3]. Briefly, after a 9-day adaptation period to the new diet, faeces were collected over a 5-day period twice daily (at 08:30 and 16:30) and frozen at -20°C. After the collection period had ended, all faeces from each dog were oven-dried at 70°C for 3 days, and then analysed in-house. The gross energy (GE) of food and faeces were measured by adiabatic bomb calorimetry, and metabolisable energy (ME) was then calculated using the following equation:

$$\text{ME}\;\left\{\text{Kcal}/\text{g}\right\}=\frac{\left[\left(\text{GE}\;\text{in}\;\text{food}\;\text{eaten}\left\{\text{Kcal}\right\}-\text{GE}\;\text{in}\;\text{faeces}\left\{\text{Kcal}\right\}\right)-\left(\text{CP}\;\text{in}\;\text{food}\;\text{eaten}\left\{\text{g}\right\}–\text{CP}\;\text{in}\;\text{faeces}\left\{\text{g}\right\}\right)\times \text{correction}\;\text{factor}\right]}{\left[\text{Food}\;\text{eaten}\left\{\text{g}\right\}\right]}$$

The correction factors used for energy lost in the urine were 1.25 and 0.86 Kcal per gram for protein for cats and dogs, respectively [7]. To enable comparison with ME estimated from predictive equations, all results were converted to Kcal per kg by multiplying by 1000.

Proximate analysis was performed according to standard methods (moisture: internal method LSE_MO_016_v3 adapted from ISO6496, NFENISO6540, NFENISO712, NFV04-401; proteins: internal method LSE_MO_018_v2 adapted from NFENISO16634-1; fat: process B, European regulation (CE) N°152/2009 by extraction and filtration; minerals: European regulation (CE) N°152/2009 by calcination). Weende’s method [23] was used to measure CF content, whilst Prosky’s method [24] was used for TDF.

### Prediction of energy content

For each dietary trial, ME was calculated using a range of predictive equations including species-specific equations. The first was an equation used MA factors [6,7], and was the same for both dog and cat food):

ME{Kcal/100g}=(3.5×CP%)−(8.5×F%)−(3.5×C%)

Carbohydrate content (C%) was estimated by calculating the nitrogen-free extract (NFE), whereby the percentages of CP, F, CF, moisture (M), and ash are subtracted from 100%. To enable comparison with other methods, results were then converted to Kcal per kg by multiplying by 10. The next predictive equations tested were those recommended in the NRC in 2006 using either CF (NRC 2006_cf_ equation) or TDF (NRC 2006_tdf_ equation) [2]. For dog food, the equations used were as follows:

$$\text{ME}\;\text{in}\;\text{dog}\;\text{food}\left\{\text{Kcal}/100\text{g}\right\}=\text{GE}\left\{\text{Kcal}/100\text{g}\right\}\times \left(\frac{91.2-\left[1.43\times \text{CF}\left\{\text{\%}\right\}\right]}{100}\right)-\left(1.04\times \text{CP}\left\{\text{\%}\right\}\right)$$

$$\text{ME}\;\text{in}\;\text{dog}\;\text{food}\left\{\text{Kcal}/100\text{g}\right\}=\text{GE}\left\{\text{Kcal}/100\text{g}\right\}\times \left(\frac{96.6-\left[0.95\times \text{TDF}\left\{\text{\%}\right\}\right]}{100}\right)-\left(1.04\times \text{CP}\left\{\text{\%}\right\}\right)$$

For cat food, the equations used were as follows:

$$\text{ME}\;\text{in}\;\text{cat}\;\text{food}\left\{\text{Kcal}/100\text{g}\right\}=\text{GE}\left\{\text{Kcal}/100\text{g}\right\}\times \left(\frac{87.9-\left[0.88\times \text{CF}\left\{\text{\%}\right\}\right]}{100}\right)-\left(0.77\times \text{CP}\left\{\text{\%}\right\}\right)$$

$$\text{ME}\;\text{in}\;\text{cat}\;\text{food}\left\{\text{Kcal}/100\text{g}\right\}=\text{GE}\left\{\text{Kcal}\right\}\times \left(\frac{95.6-\left[0.89\times \text{TDF}\left\{\text{\%}\right\}\right]}{100}\right)-\left(0.77\times \text{CP}\left\{\text{\%}\right\}\right)$$

Again, results were converted to Kcal per kg by multiplying by 10. Finally, ME was also estimated using equations recently reported (Hall equations) [6]. For dog food, the equation was as follows:

ME{Kcal/kg}=575+(0.8166×GE{Kcal})+(12.086×F%)−(52.766×CF%)−(20.616×CP%)−(6.076×M%)

For cat food, the equation was as follows:

ME{Kcal/kg}=2541+(0.9236×GE{Kcal})+(14.686×F%)−(44.316×CF%)−(4.216×CP%)−(4.806×M%)

Finally, in all methods requiring GE (NRC 2006_tdf_, NRC 2006_cf_ and Hall equations), comparisons were made between the performance of each method when GE was either measured (as described above) or predicted using the following equation [2]:

GE{kcal/kg}=(57×CP{g})+(94×F{g})+(41×[NFE{g}+CF{g}])

### Statistical analysis

Data were analysed using different statistical software packages (JMP^®^ version 14.2.0, SAS Institute Inc.; Prism for Mac version 8.10, Graph Pad Software Inc.). Given the number of statistical comparisons, a modified Bonferroni correction was applied within each ‘family’ of tests, e.g. for the same type of statistical test within a single diet type (dry or wet) within a single species (dog or cat), such that statistical significance was only considered when *P* <0.007. The Friedman test was used to compare measured and predicted ME for each diet type; with post-hoc comparisons made with Steel’s test, whereby the predicted ME from each equation was compared against measured ME. Deming regression was used to compare associations between ME predicted with each equation and ME measured in the feeding trial [20]. Acceptability was further assessed by constructing Bland-Altman plots [21]. For each plot, average bias and 95% limits of agreement were calculated, with results analysed further by with a 1-sample T test (to assess the significance of any bias between measured and predicted ME), and simple linear regression analysis (comparing mean of and differences between the two methods) to assess for possible proportional errors.

## Results

### Feeding trials and diets included in final analysis

A total of 547 canine feeding trials using dry expanded food were eligible for inclusion. Of these, there were sufficient data available in 315 trials to calculate ME by the NRC 2006_tdf_, NRC 2006_cf_, and MA equations. Of these, sufficient data were available from 182 trials to calculate ME using the Hall equation (S2 File). In order to ensure a fair comparison amongst methods, this final dataset was used in all statistical analyses. A total of 332 feline feeding trials using dry expanded food were eligible for inclusion. Of these, there were sufficient data available in 164 trials to calculate ME by the NRC 2006_tdf_, NRC 2006_cf_, and MA equations. Of these, sufficient data were available from 104 trials to calculate ME using the Hall *et al* equation (S3 File). A total of 26 canine feeding trials on wet food were also eligible for inclusion, and sufficient data were available from 25 of these to calculate ME by all methods (S4 File). A total of 29 feline feeding trials on wet food were also eligible for inclusion, and sufficient data were available from 24 of these to calculate ME by all methods (S5 File). Summary statistics of the guaranteed analyses of the canine and feline foods that were used in the feeding trials finally included in the study are given in Tables 1 and 2, respectively.

**Table 2:** Composition of feline foods assessed in the study.

	Dry Food^1^ (n = 104)				Wet food^1^ (n = 24)			
	Mean	SD	Median	Range	Mean	SD	Median	Range
Measured GE^2^	4825	236.2	4773	4399–5464	1138	146.9	1140	870–1450
Predicted GE^2^	4927	228.6	4897	4517–5556	1173	179.0	1155	919–1644
Crude protein (%)	34.0	3.90	33.9	25.8–46.8	9.1	1.82	8.6	6.4–14.2
Crude fat (%)	15.4	4.24	15.1	8.0–25.1	4.7	1.79	4.5	1.5–8.6
Total dietary fibre (%)	9.2	3.73	9.6	2.5–24.8	1.8	0.60	1.7	0.7–3.0
Crude fibre (%)	4.3	2.42	4.4	0.7–14.1	1.0	0.20	1.0	1.0–2.0
Ash (%)	6.9	1.07	6.9	4.4–9.3	1.6	0.49	1.4	0.9–2.9
Moisture (%)	6.2	1.03	6.1	3.2–8.4	79.4	2.32	79.9	72.6–83.1

^1^ Values reported are on an as fed basis.

^2^GE: gross energy content of food in Kcal, determined either by measurement [3] or prediction [2].

### Measured and predicted ME in dry canine diets

In the 182 available trials on canine dry food, mean ±standard deviation (SD) measured ME was 3897 ±313.6 Kcal/kg (16305 ±1312.3 kJ/kg). Using the Friedman test, significant differences were identified amongst methods used to predict ME (Table 3, *P*<0.001). On average, predictions using the MA equation (*P*<0.001), NRC 2006_cf_ (using both predicted [*P* = 0.008] and measured [*P*<0.001] GE), and Hall equations (using measured GE, *P* = 0.003) all underestimated measured ME, whilst predictions using the NRC 2006_tdf_ (using predicted GE, *P*<0.001) and Hall (using predicted GE, *P* = 0.003) equations both overestimated measured GE. However, there was no difference between measured ME and average predictions made by the NRC 2006_tdf_ equation (using measured GE, *P* = 1.000).

**Table 3:** Measured and predicted metabolisable energy content of canine foods assessed in the study.

	Dry Food^1^ (n = 182)					Wet food^1^ (n = 25)				
	Mean	SD	Median	Range	P-value^2^	Mean	SD	Median	Range	P-value^2^
Measured ME^3^	3897	313.6	3969	2821–4532	---	1039	261.6	1010	535–1703	---
Modified Atwater^4^	3705	240.2	3761	2946–4340	<0.001	925	249.6	906	485–1663	<0.001
NRC 2006_cf_^5^										
Predicted GE^6^	3871	323.6	3945	2655–4527	0.008	1128	254.5	1084	634–1849	<0.001
Measured GE^6^	3816	308.0	3864	2702–4485	<0.001	1151	229.4	1101	651–1742	<0.001
NRC 2006_tdf_^7^										
Predicted GE^6^	3953	324.0	4039	2773–4639	<0.001	1034	271.2	1014	562–1834	0.996
Measured GE^6^	3901	304.7	3967	2800–4596	1.000	1056	252.0	1016	584–1725	0.999
Hall *et al*^8^										
Predicted GE^6^	3925	298.2	3991	2853–4622	0.003	962	284.8	930	431–1785	<0.001
Measured GE^6^	3875	298.2	3991	2853–4622	0.003	983	259.2	946	447–1687	0.003

^1^ Reported values are kcal per 1000g of food on an as fed basis.

^2^ Reported P-values are those from the Friedman test, comparing predicted with measured ME content.

^3^ ME: metabolisable energy content of food.

^4^ ME predictions calculated using modified Atwater factors [6,7].

^5^ ME predictions calculated using species-specific equations based upon crude fibre [2].

^6^ GE: gross energy content of food, determined either by measurement [3] or prediction [2].

^7^ ME predictions calculated using species-specific equations based upon total dietary fibre [2].

^8^ ME predictions calculated using species-specific equations reported by Hall *et al*. [16].

**Fig 1:**
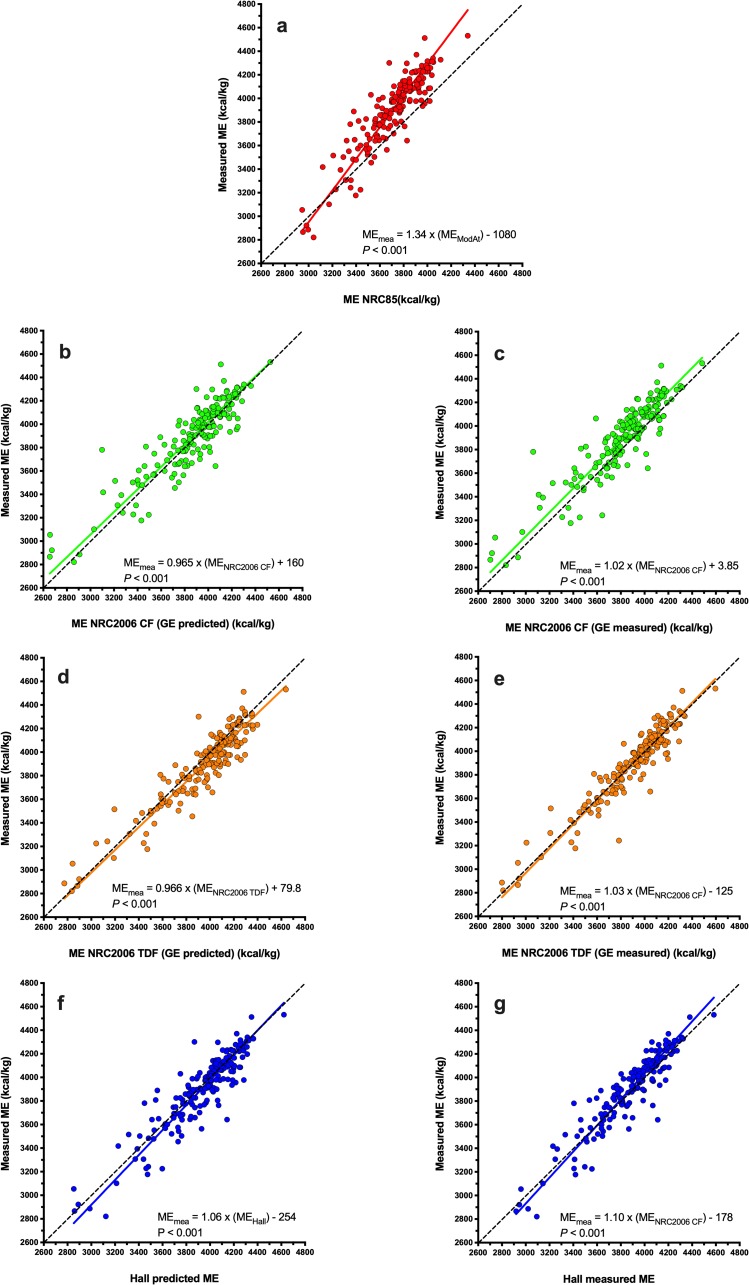
Deming regression plot depicting the relationship between measured and predicted metabolisable energy (ME) content in dry canine diets. The points represent results for individual foods, the solid line depicts the trendline from Deming regression, and the dotted black line represents the line of equivalence (where each unit increase in predicted ME equates to the same increase in measured ME. Different figures represent predictions made with MA (red, a), NRC 2006_cf_ (green, b: predicted GE; c: measured GE), NRC 2006_tdf_ (orange; d: predicted GE; e: measured GE), and Hall equations (blue; f: predicted GE; g: measured GE).

**Fig 2:**
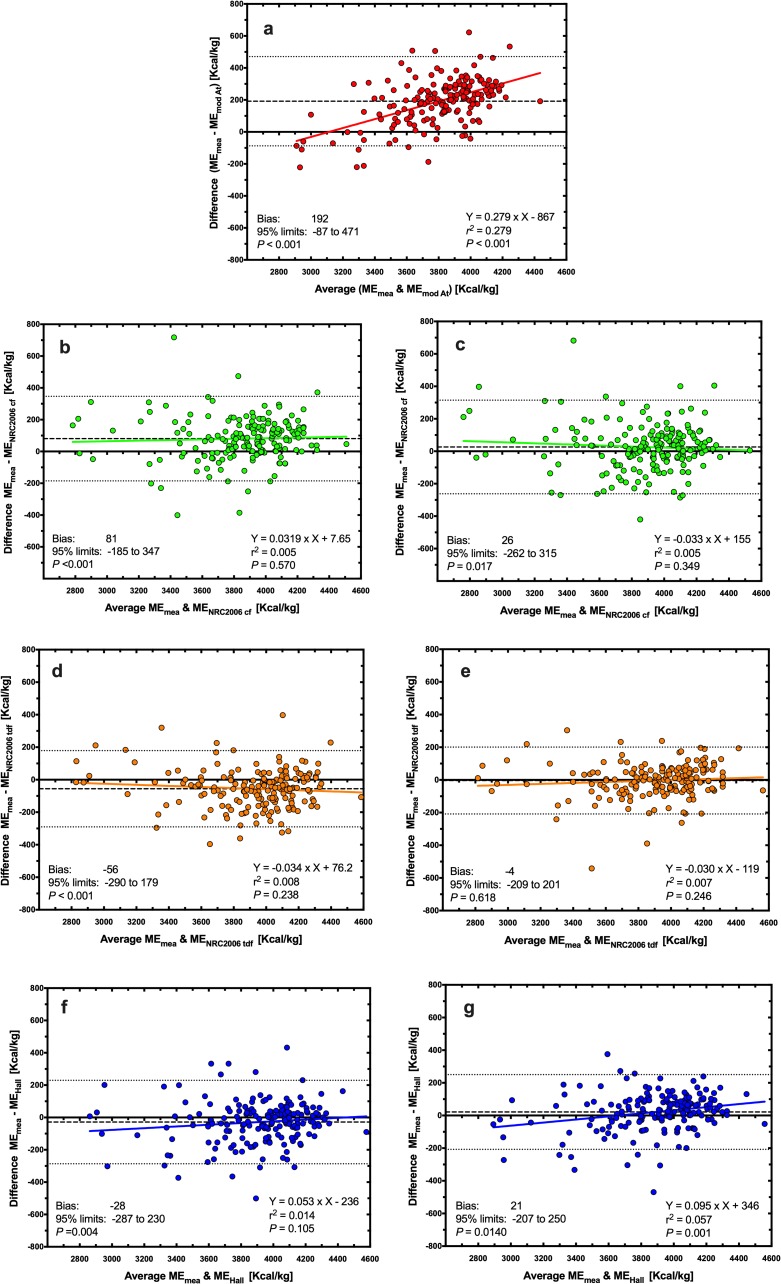
Bland-Altman plot depicting the relationship between measured and predicted metabolisable energy (ME) content in dry canine diets. The y- and x-axes depict the difference between and the average of the measured and predicted ME content of dry canine diets, respectively. The points represent results for individual foods, the dashed back line depicts the average bias, the dotted black lines depict the 95% confidence limits to the agreement, and the solid coloured line MA Atwater (red, a), NRC 2006_cf_ (green; b: predicted GE, c: measured GE), NRC 2006_tdf_ (orange; d: predicted GE; e: measured GE), and Hall equations (blue; f: predicted GE; g: measured GE).

Using Deming regression analysis, predicted ME was positively associated with measured ME whatever the equation used (Fig 1, *P*<0.001 for all). Predictions using the MA equation differed most (ME_measured_ = 1.34 × ME_MA_ -1080; Fig 1A), whilst predictions using the NRC 2006_tdf_ and NRC 2006_cf_ equations (both with measured GE) differed least (ME_measured_ = 1.03 × ME_NRC20006 TDF_ -125, *P*<0.001, Fig 1C; ME_measured_ = 1.02 × ME_NRC20006 CF_ + 3.85, *P*<0.001, Fig 1E) from measured ME. Associations between predicted and measured ME were explored further using Bland-Altman plots (Fig 2). Agreement was worst for the MA equations where there was both a significant bias and a proportional error across the data range, with predictions of ME being underestimated most in diets with the greatest ME content (Fig 2A). Overall agreement was better for both the NRC 2006_cf_ and Hall equations (using either measured or predicted GE) although either significant bias or proportional errors were seen (Fig 2B, 2C, 2F and 2G). The best agreement with measured ME were for predictions made with the NRC 2006_tdf_ equation using measured GE, where there was no significant bias or proportional error, and the 95% limits of agreement were narrowest (Fig 2E).

### Measured and predicted ME in dry feline diets

In the 104 available trials on feline dry food, mean ±SD measured ME was 3935 ±351.2 Kcal/kg (16464 ±1469.2 kJ/kg). Using the Friedman test, significant differences were identified amongst methods used to predict ME (Table 4, *P*<0.001). Average predictions using the MA equation (*P*<0.001), NRC 2006_cf_ equation (using both predicted and measured GE, *P*<0.001 for both), and Hall equation (using measured GE, *P*<0.001) underestimated measured ME, whilst predictions using the NRC 2006_tdf_ equation (using predicted GE, *P*<0.001) overestimated measured ME. However, there was no difference between measured ME and average predictions made by both the NRC 2006_tdf_ (using measured GE, *P* = 0.998) and Hall (using either predicted GE, *P* = 0.999) equations.

**Table 4:** Measured and predicted metabolisable energy (ME) content of feline foods assessed in the study.

	Dry Food^1^ (n = 104)					Wet food^1^ (n = 24)				
	Mean	SD	Median	Range	P-value^2^	Mean	SD	Median	Range	P-value^2^
Measured ME^3^	3935	351.2	3903	3089–4654	---	938	140.2	955	686–1186	---
Modified Atwater^4^	3666	277.3	3649	3034–4318	<0.001	819	146.7	823	611–1158	<0.001
NRC 2006_cf_^5^										
Predicted GE^6^	3874	271.4	3854	3195–4538	<0.001	1000	145.6	999	793–1405	<0.001
Measured GE^6^	3792	275.9	3763	3202–4458	<0.001	983	126.4	986	750–1239	<0.001
NRC 2006_tdf_^7^										
Predicted GE^6^	4023	328.9	3977	3078–4745	<0.001	952	161.9	955	730–1313	0.003
Measured GE^6^	3938	331.1	3887	3085–4668	0.998	927	130.7	951	688–1195	0.891
Hall *et al*^8^										
Predicted GE^6^	3930	341.3	3900	3124–4774	0.999	892	164.2	886	664–1279	0.436
Measured GE^6^	3935	351.2	3903	3089–4654	<0.001	874	144.1	881	619–1175	<0.001

^1^ Reported values are Kcal per 1000g of food on an as fed basis.

^2^ Reported P-values are those from the Friedman test, comparing predicted with measured ME content.

^3^ ME: metabolisable energy content of food.

^4^ ME predictions calculated using modified Atwater factors [6,7].

^5^ ME predictions calculated using species-specific equations based upon crude fibre [2].

^6^ GE: gross energy content of food, determined either by measurement [3] or prediction [2].

^7^ ME predictions calculated using species-specific equations based upon total dietary fibre [2].

^8^ ME predictions calculated using species-specific equations reported by Hall *et al*. [16].

**Fig 3:**
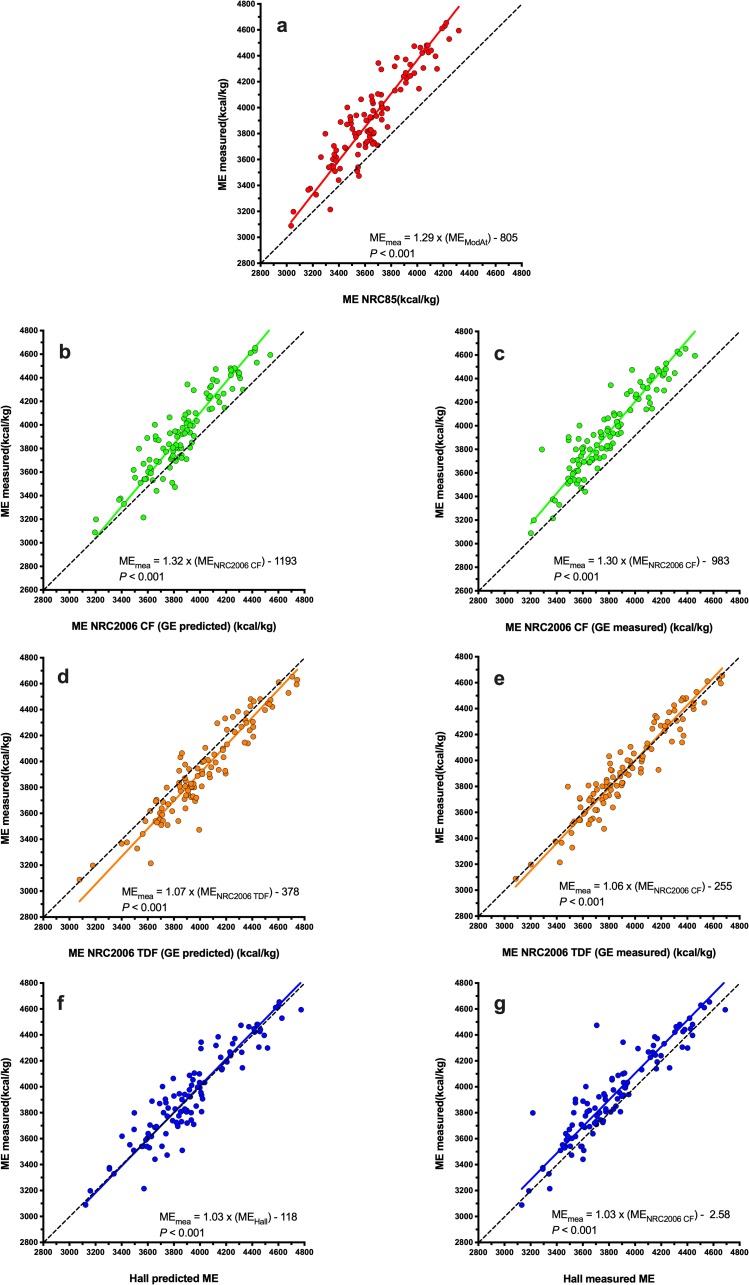
Deming regression plot depicting the relationship between measured and predicted metabolisable energy (ME) content in dry feline diets. The points represent results for individual foods, the solid line depicts the trendline from Deming regression, and the dotted black line represents the line of equivalence (where each unit increase in predicted ME equates to the same increase in measured ME. Different figures represent predictions made with MA (red, a), NRC 2006_cf_ (green, b: predicted GE; c: measured GE), NRC 2006_tdf_ (orange; d: predicted GE; e: measured GE), and Hall equations (blue; f: predicted GE; g: measured GE).

**Fig 4:**
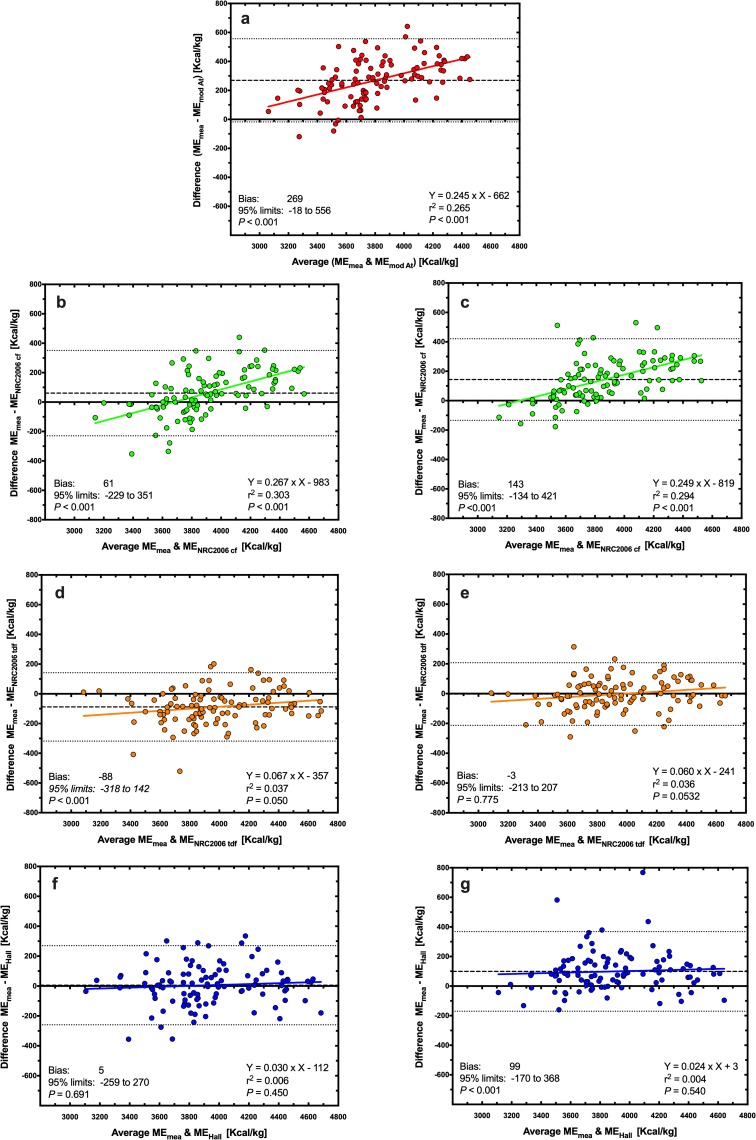
Bland-Altman plot depicting the relationship between measured and predicted metabolisable energy (ME) content in dry feline diets. The y- and x-axes depict the difference between and the average of the measured and predicted ME content of dry feline diets, respectively. The points represent results for individual foods, the dashed back line depicts the average bias, the dotted black lines depict the 95% confidence limits to the agreement, and the solid coloured line represents the trendline from linear regression analysis. Different figures represent predictions made with MA (red, a), NRC 2006_cf_ (green; b: predicted GE; c: measured GE), NRC 2006_tdf_ (orange; d: predicted GE; e: measured GE), and Hall equations (blue; f: predicted GE; g: measured GE).

Using Deming regression analysis, predicted ME was positively associated with measured ME whatever the equation used (Fig 3, *P*<0.001 for all). Predictions using the MA equation (ME_measured_ = 1.29 × ME_MA_ − 805; Fig 3A) and the NRC 2006_cf_ equation using both predicted (ME_measured_ = 1.32 × ME_NRC2006 CF_− 1193; Fig 3B) and measured (ME_measured_ = 1.30 × ME_NRC2006 CF_− 983, Fig 3C) GE differed most from measured ME, whilst predictions using the NRC 2006_tdf_ equation using measured GE (ME_measured_ = 1.06 × ME_NRC2006 TDF_− 255; Fig 3E) and the Hall equation using predicted GE (ME_measured_ = 1.03 × ME_Hall_– 118; Fig 3F) differed least from measured ME. When associations between predicted and measured ME were explored further using Bland-Altman plots (Fig 4), agreement was worst for both MA (Fig 4A) and NRC 2006_cf_ equations using both predicted (Fig 4B) and measured (Fig 4C) GE, with significant bias and also proportional errors across the data range. Overall agreement was better for the Hall equation using predicted GE (Fig 4F) where there was no significant bias or proportional error. However, agreement was best for the NRC 2006_tdf_ equation using measured GE where, in addition to there being no significant bias or proportional error, the 95% limits of agreement were narrowest (Fig 4E).

### Measured and predicted ME in wet canine diets

In the 25 available trials assessing canine wet food, mean ±SD was measured ME was 1039 ±261.6 Kcal/kg (4347 ±1094.6 kJ/kg). Using the Friedman test, significant differences were identified amongst methods used to predict ME (Table 2, *P*<0.001). On average, predictions using the MA (*P*<0.001) and Hall (using measured [*P* = 0.003] and predicted [*P*<0.001] GE) equations underestimated measured ME, whilst predictions using the NRC 2006_cf_ equation (using both predicted and measured GE) overestimated measured ME (*P*<0.001 for both). In contrast, there was no difference between measured ME and average predictions made by the NRC 2006_tdf_ equation using both predicted (*P* = 0.996) and measured (*P* = 0.999) GE.

**Fig 5:**
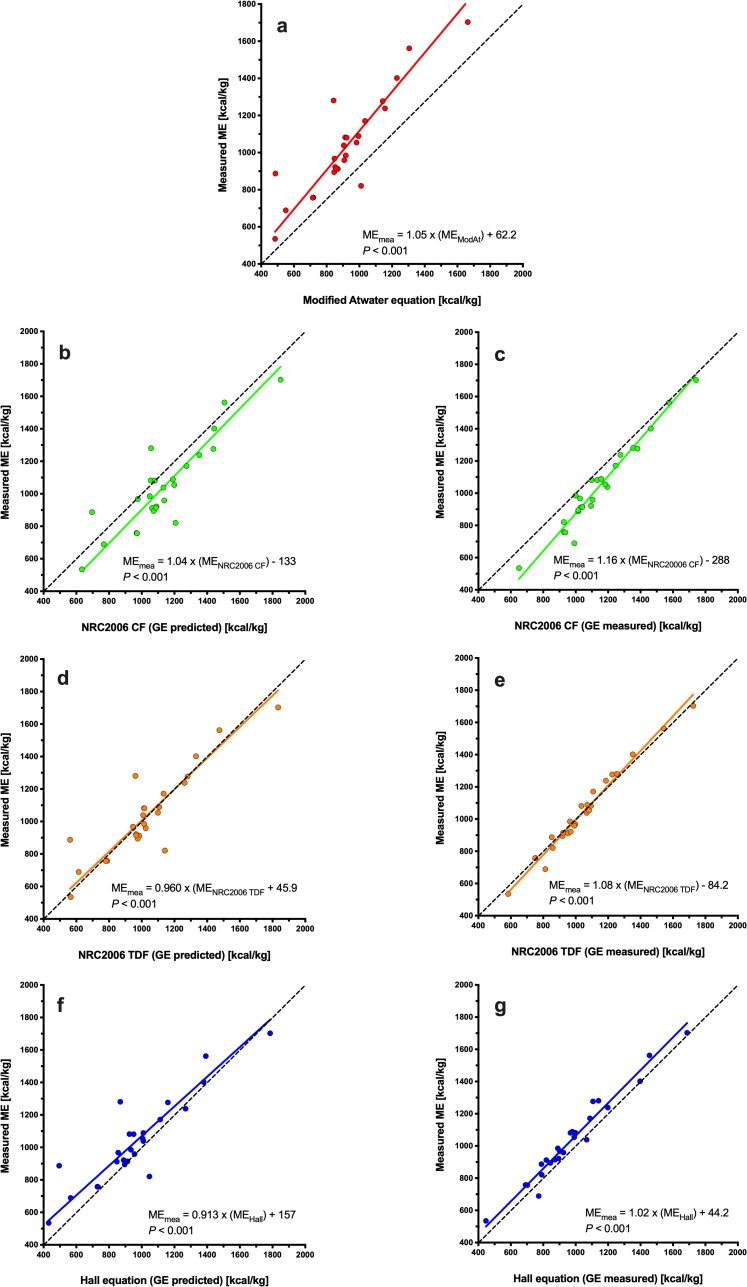
Deming regression plot depicting the relationship between measured and predicted metabolisable energy (ME) content in wet canine diets. The points represent results for individual foods, the solid line depicts the trendline from Deming regression, and the dotted black line represents the line of equivalence (where each unit increase in predicted ME equates to the same increase in measured ME. Different figures represent predictions made with MA (red, a), NRC 2006_cf_ (green; b: predicted GE; c: measured GE), NRC 2006_tdf_ (orange; d: predicted GE; e: measured GE), and Hall equations (blue; f: predicted GE; g: measured GE).

**Fig 6:**
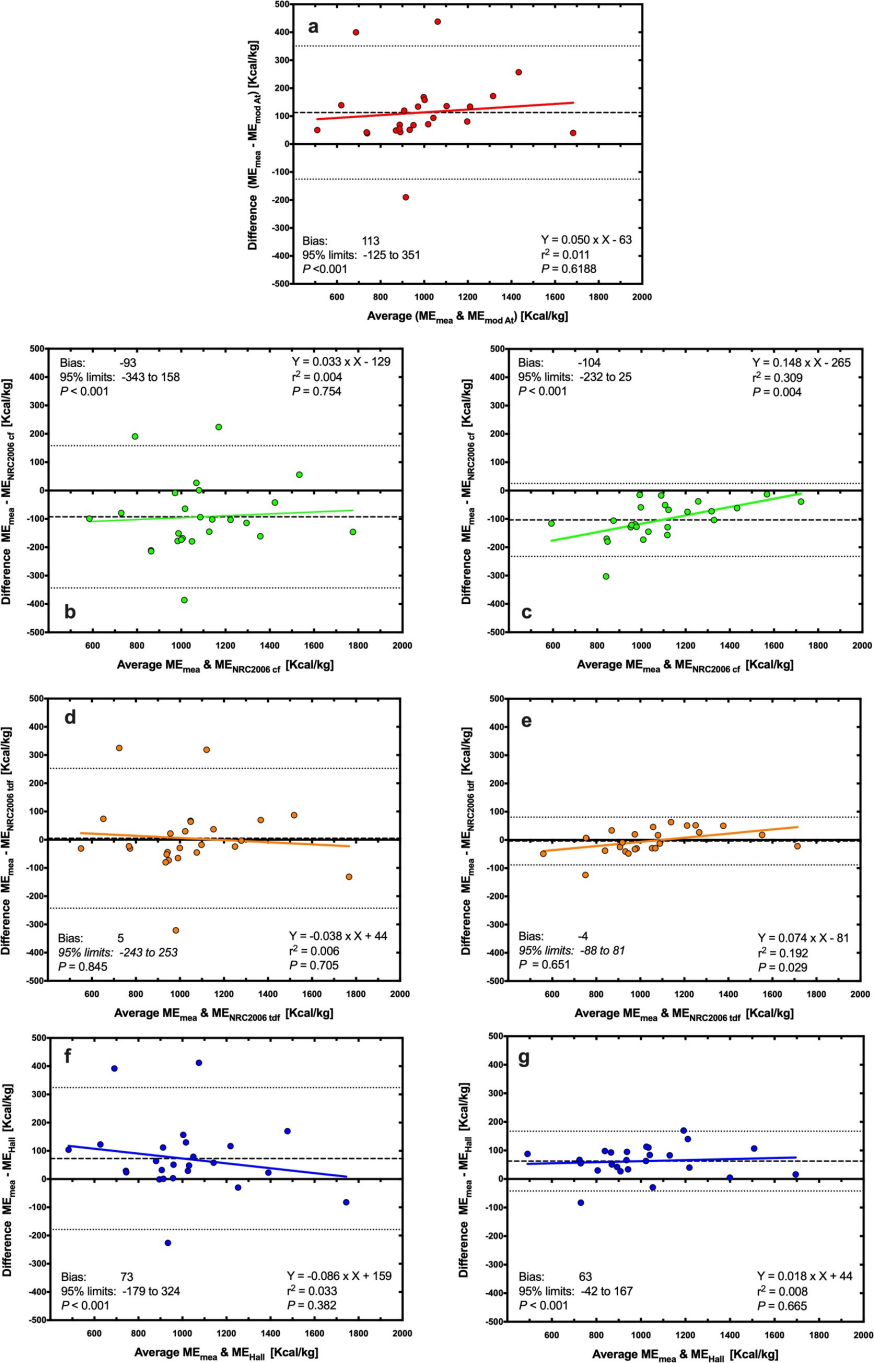
Bland-Altman plot depicting the relationship between measured and predicted metabolisable energy (ME) content in wet canine diets. The y- and x-axes depict the difference between and the average of the measured and predicted ME content of dry canine diets, respectively. The points represent results for individual foods, the dashed back line depicts the average bias, the dotted black lines depict the 95% confidence limits to the agreement, and the solid coloured line represents the trendline from linear regression analysis. Different figures represent predictions made with MA (red, a), NRC 2006_cf_ (green; b: predicted GE; c: measured GE), NRC 2006_tdf_ (orange; d: predicted GE; e: measured GE), and Hall equations for dogs (blue; f: predicted GE; g: measured GE).

Using Deming regression analysis, predicted ME was positively associated with measured ME whatever the equation used (Fig 5, *P*<0.001 for all). Predictions using the MA equation (ME_measured_ = 1.05 × ME_MA_ + 62.2; Fig 5A), the NRC 2006_cf_ equation using measured GE (ME_measured_ = 1.16 × ME_NRC CF_ − 288; Fig 5C) and the Hall equation using predicted GE (ME_measured_ = 0.913 × ME_Hall_ + 157; Fig 5F) differed most from measured ME, whilst predictions using the NRC 2006_tdf_ equation using both predicted (ME_measured_ = 0.960 × ME_NRC20006 TDF_ − 45.9, *P*<0.001, Fig 5d) and measured (ME_measured_ = 1.08 × ME_NRC20006 TDF_− 84.2, *P*<0.001, Fig 5E) GE, differed least from measured ME. Associations between predicted and measured ME were explored further using Bland-Altman plots (Fig 6). Agreement was worst for both the MA (Fig 6A) and NRC 2006_cf_ equations using either predicted (Fig 6B) or measured (Fig 6C) GE, all of which had both significant bias and proportional error. Overall agreement was better for the Hall equation (using either measured or predicted GE) although either significant bias or proportional errors were still evident (Fig 6B, 6C, 6F and 6G). Neither significant bias nor proportional error was evident for predictions made with the NRC 2006_tdf_ equation using predicted GE, although the 95% limits of agreement were relatively wide (Fig 6D). Overall, the best agreement with measured ME was seen with the NRC 2006_tdf_ equation where there was no significant bias or proportional error, and the 95% limits of agreement were narrowest (Fig 6E).

### Measured and predicted ME in wet feline diets

In the 24 available trials assessing feline wet food, mean ±SD measured ME was 938 ±140.2 Kcal/kg (3925 ±568.8 kJ/kg). Using the Friedman test, significant differences were identified amongst methods used to predict ME (Table 4, *P*<0.001). On average, predictions using the MA (*P*<0.001) and Hall equations (using measured GE, *P*<0.0001) underestimated measured ME, whilst predictions using the NRC 2006_cf_ (using predicted and measured GE, *P*<0.001 for both) and NRC 2006_tdf_ (using predicted GE, *P* = 0.003) equations overestimated measured ME. In contrast, there was no difference between measured ME and average predictions made by either the NRC 2006_tdf_ equation using both measured (*P* = 0.891) GE and the Hall equation using predicted GE (*P* = 0.436).

**Fig 7:**
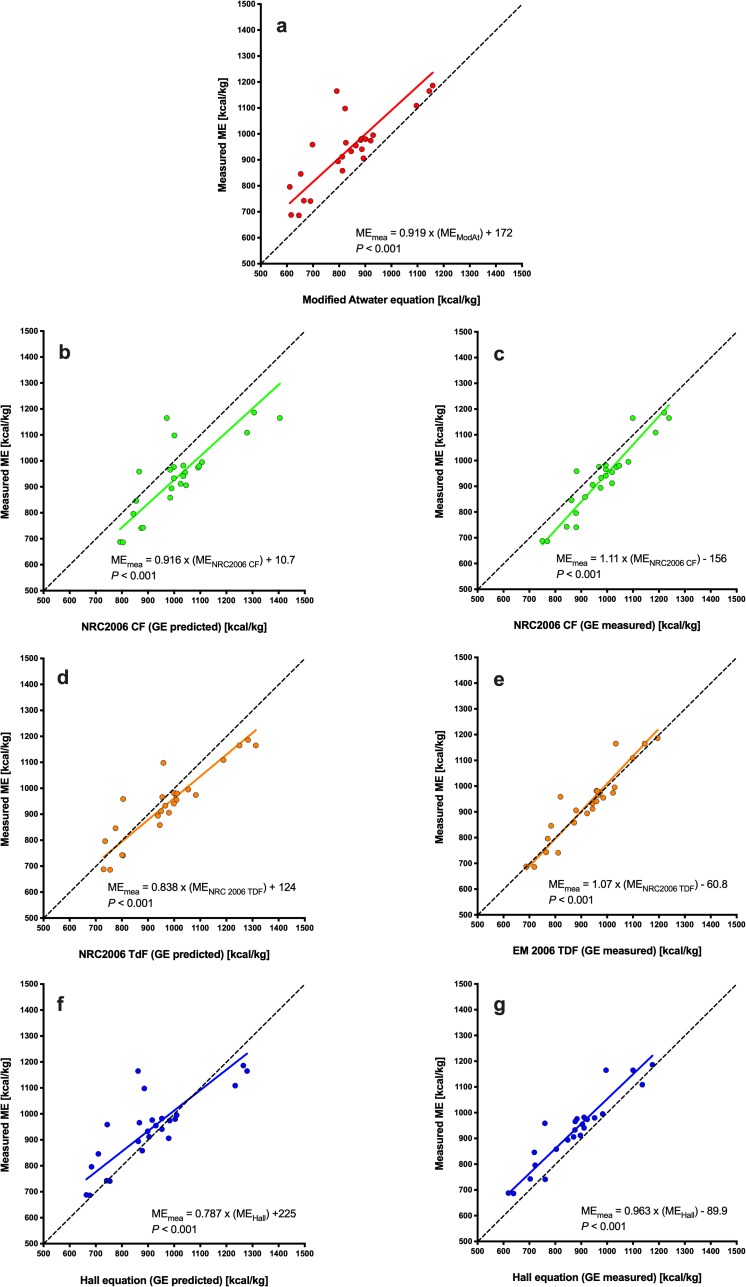
Deming regression plot depicting the relationship between measured and predicted metabolisable energy (ME) content in wet feline diets. The points represent results for individual foods, the solid line depicts the trendline from Deming regression, and the dotted black line represents the line of equivalence (where each unit increase in predicted ME equates to the same increase in measured ME. Different figures represent predictions made with modified Atwater (red, a), NRC 2006_cf_ (green; b: predicted GE; c: measured GE), NRC 2006_tdf_ (orange; d: predicted GE; e: measured GE), and the Hall equation for cats (blue; f: predicted GE; g: measured GE).

**Fig 8:**
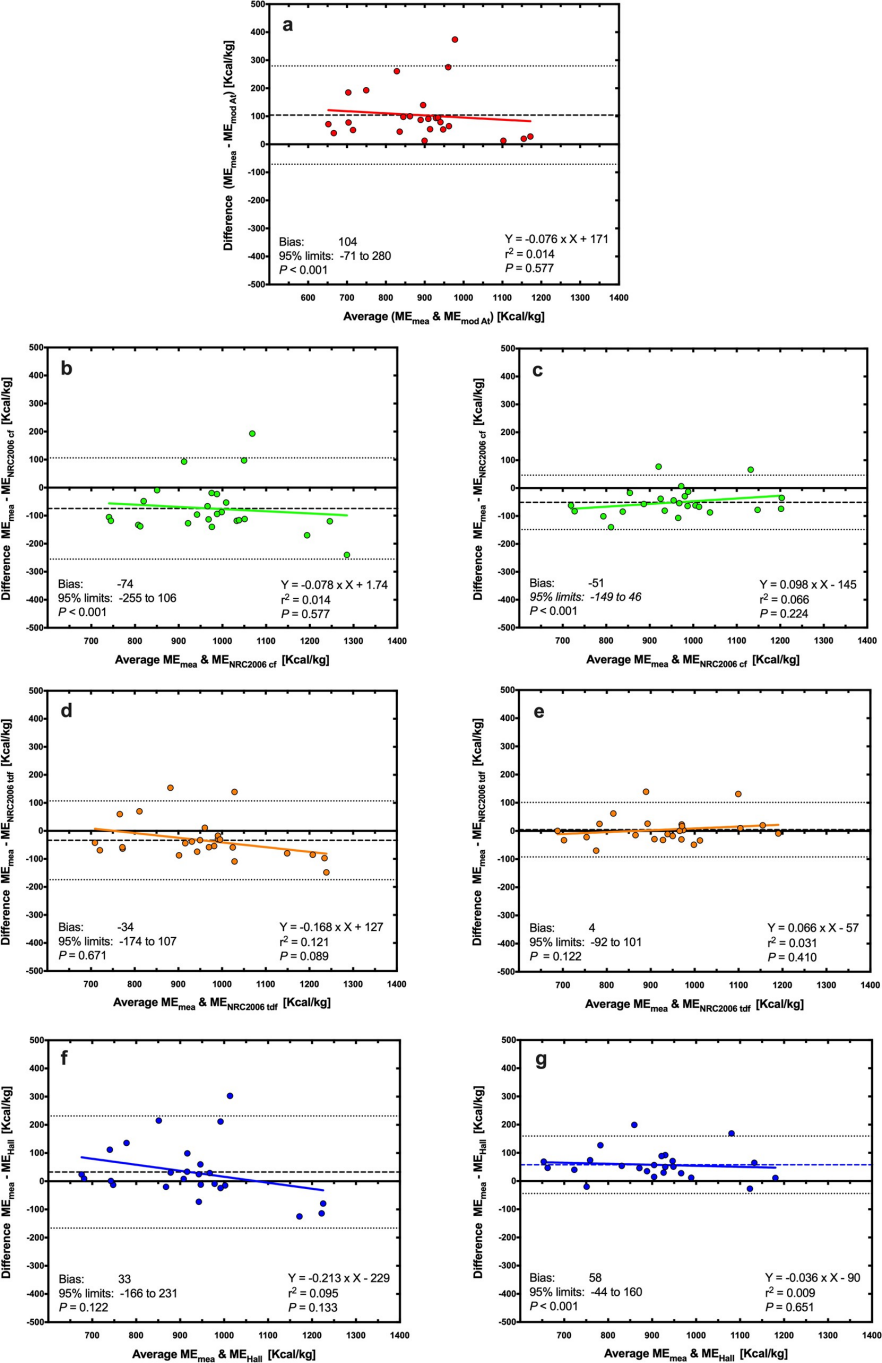
Bland-Altman plot depicting the relationship between measured and predicted metabolisable energy (ME) content in wet feline diets. The y- and x-axes depict the difference between and the average of the measured and predicted ME content of dry canine diets, respectively. The points represent results for individual foods, the dashed back line depicts the average bias, the dotted black lines depict the 95% confidence limits to the agreement, and the solid coloured line represents the trendline from linear regression analysis. Different figures represent predictions made with modified Atwater (red, a), NRC 2006_cf_ (green; b: predicted GE; c: measured GE), NRC 2006_tdf_ (orange; d: predicted GE; e: measured GE), and the Hall equation for cats (blue; f: predicted GE; g: measured GE).

Using Deming regression analysis, predicted ME was positively associated with measured ME whatever the equation used (Fig 7, *P*<0.001 for all). Predictions using the MA (ME_measured_ = 0.919 × ME_MA_ + 172; Fig 7A) and the Hall (ME_measured_ = 0.787 × ME_Hall_ + 225; Fig 7F) equations differed most from measured ME, whilst predictions using the NRC 2006_tdf_ equation using measured GE (ME_measured_ = 1.07 × ME_NRC2006 TDF_− 60.8, *P*<0.001, Fig 7E) differed least from measured ME. Associations between predicted and measured ME were explored further using Bland-Altman plots (Fig 8). Agreement was worst for both the MA (Fig 8A) and NRC_2006cf_ equations using either predicted (Fig 8B) or measured (Fig 8C) GE, with all methods having significant bias. Overall agreement was better for the Hall equation using predicted GE (Fig 8F) although the 95% limits of agreement were relatively wide. Agreement was also good for the Hall equation using measured GE (Fig 8G), with narrow 95% limits of agreement, although significant proportional error was evident. Neither significant bias nor proportional error was evident for predictions made with the NRC 2006_tdf_ equation using predicted GE, although the 95% limits of agreement were relatively wide (Fig 8D). Overall, the best agreement with measured ME was seen with the NRC 2006_tdf_ equation using measured GE where there was no significant bias or proportional error, and the 95% limits of agreement were narrowest (Fig 8E).

## Discussion

In the current study, we have analysed data from 355 feeding studies in dogs and cats, which were performed according to AAFCO guidelines [3] over a period of 5 years. Our aim was to compare accuracy of various predictive equations for estimating ME [2,4–9,12,13,16]. Overall, the MA equation performed worst of all, the NRC 2006_cf_ and Hall equations were intermediate in performance, whilst the NRC 2006_tdf_ equations performed best especially when using measured rather than predicted GE. These results clarify and extend previous work assessing performance of predictive equations for ME content in dog and cat food [7,8,10,15], emphasising the superiority of the NRC 2006_tdf_ equations over all other methods [13].

Predicting ME content of food using the MA equation is the most established method, and was the method originally recommended by NRC [9]. Its main advantage is simplicity and the fact that the same equation can be applied both to dogs and cats [9]. However, consistent with previous studies, this method performed worst of all in the current study: although there was a significant association between measured ME and ME predicted by the MA equation, a systematic bias existed for every food type, meaning that this equation tended to underestimate measured ME. Average bias was equivalent to a ~5% underestimate for an average dry food, but closer to a ~11% for an average wet food. Further, as highlighted by the 95% limits of agreement, substantial discrepancies existed for individual foods; for example, ME predictions for dry canine food ranged from an 8% overestimate to a 16% underestimate, whilst predictions for wet dog food ranged from a 21% overestimate to a 58% underestimate. Further, a proportional error also existed for ME predictions on dry dog and cat food whereby, as the greater the measured ME of the food, the greater the underestimate from the prediction. Such underestimates of ME are concerning since they might lead to overfeeding. Given the results of the current study, nutritionists and veterinary clinicians should consider other methods for predicting the ME content of dog and cat food whenever possible.

In the most recently-published study assessing predictive equations for ME in dog and cat food, the NRC 2006_cf_ equation performed as well as, but not better than, the MA equation [16]. These results contrast with the results of the current study where the predictions from the NRC 2006_cf_ equation were superior to those of the MA equation [16]. The reason for this difference is likely due to the fact that predicted GE was used in the previous study [16]. In the current study, the NCR 2006_cf_ equation performed better when using measured GE rather than predicted GE; with predicted GE, performance of this equation was marginally better than the MA equation in terms of systematic bias, but there was still a marked proportional error, when predicting ME content of feline dry food especially. Using measured, rather than predicted, GE improved the accuracy of predictions better, with narrower 95% limits of agreement (especially for wet food) but did not totally resolve the issues with proportional bias.

Hall *et al*. also generated new equations which performed better than both the MA and NRC 2006_cf_ equations [16]. As highlighted above, a limitation was that the same dataset was used both to generate the predictive equations and test them. Therefore, the current study was an opportunity to assess the performance of the equations proposed by this study with an independent dataset. Further, whilst the previous study only used measured GE with the new equations [16], performance of the new equations was tested using both measured and predicted GE. Interestingly, the effect of substituting measured for predicted GE was variable depending upon the type of food; performance deteriorated when predicting the ME of both canine and feline wet food, with the 95% limits of agreement becoming wider, whilst performance in predicting measured ME of dry food was similar or marginally better. As with the Hall *et al*. study [16], the new equations predicted the ME of pet food better than did the MA equation whether predicted or measured GE was used. Performance was also better than with the NRC 2006_cf_ equation when using predicted GE. However, when measured GE was also used with the NRC 2006_cf_ equations, the Hall equation was no longer superior. Thus, the previously-reported advantage of the Hall equations over the 2006_cf_ equations is more likely to be the result of their use of measured GE in the NRC 2006_cf_ equations, rather than a superiority of a predictive equation that includes terms of moisture, protein, fat, and fibre [16].

Of all methods tested, the NRC 2006_tdf_ equations performed best, especially when using measured GE, with the least average bias and narrowest 95% limits of agreement. Further, although a proportional error was observed when predicting ME, the effect was minimal across the range of ME content in the wet foods studied. Therefore, we strongly recommend the use of this equation by nutritionists and veterinarians whenever possible. Further, although use of these equations is already recommended by FEDIAF, other regulatory bodies should consider making the same recommendation, ensuring globally-compatible approaches to ME reporting. Of course, one challenge is that many food companies neither measure TDF nor GE in their products. Consistent adoption of the NRC2006_tdf_ would encourage more companies to perform such measurements in the future. Universal use of this equation would give veterinary professionals confidence when providing feeding advice to owners regarding proprietary food. Nonetheless, it should be emphasised that this will not completely resolve all challenges in actually determining daily food portions, not least given the variability in ME required amongst individual animals, which can vary according to age, sex, neuter status, husbandry, and activity [25,26]. Therefore, any estimates of daily food intake should subsequently be adjusted according to response, for example, by adjusting the allocation to ensure that bodyweight and BCS remain stable over time. Further sources of inaccuracy include methods used to measure food portions (e.g. the use of measuring cups [27]) and feeding extra food in the form of treats and table scraps [28]. Therefore, to avoid overfeeding, owners should also be made aware of how to measure out food portions accurately (e.g. using electronic gram scales) and also about the potential impact of feeding additional food.

The study has a number of limitations that should be considered. First, although the number of available studies was large, some datasets were incomplete limiting the number of available food trials for the final analyses. Secondly, only a limited number of feeding trials were conducted on wet food during the study period meaning that conclusions about such food should be made more cautiously. Third, the studies were undertaken over a five-year period and only small numbers of animals from a single research colony; therefore, actual availability of ME in a diverse population of pet cats and dogs might be different. That said, this period was equivalent to that used for predictions made in the Hall *et al*. study [16]. Finally, although foods from a range of manufacturers were tested, these were mainly those of established pet food companies that use conventional methods such as extrusion. Recently, a number of smaller companies have been established and recipes are now more diverse, including vegetarians and grain-free options, those using novel protein sources including insects, and also those using uncooked ingredients. Such diets often differ in methods of manufacture digestibility, and the extent to which they are nutritionally complete. Therefore, it is unclear whether the findings of the current study can be extrapolated to all commercially-available diets, and further studies should be considered to confirm the study findings.

## Conclusions

In the current study we have assessed the ability of a range of predictive equations to determine the measured ME content in prepared pet foods. Predictions using the MA equation performed worst, whilst those using the NRC 2006 approach performed best, provided that TDF was used rather than CF. As reported by Hall *et al*. [16], use of measured, rather than predicted, GE improves predictions. Pet food manufacturers and regulatory bodies are strongly recommended to measure and report ME using these formulae in the future. If ME content of food is not reported on the pet food label, TDF and measured GE should be reported in order to enable veterinarians to use this equation in their own calculations in clinical practice.

## Supporting information

S1 File Study diets. Spreadsheet containing details of the diets used in the study.
(XLSX)

S2 File Metabolisable energy of dry canine diets. (XLSX)

S3 File Metabolisable energy of dry feline diets. (XLSX)

S4 File Metabolisable energy of wet canine diets. (XLSX)

S5 File Metabolisable energy of wet feline diets. (XLSX)

## References

[1] Thatcher CD, Hand MS, Remillard RL. Small animal clinical nutrition: an iterative process In: Hand MS, Thatcher CD, Remillard RL, Roudebush P, Novotny BJ, editors. Small animal clinical nutrition. 5th ed Topeka KS: Mark Morris Institute; 2010 pp. 3–21.

[2] Nutritional Research Council. Nutrient requirements of dogs and cats. Washington, DC: The National Academies Press; 2006.

[3] Association of American Feed Control Officials. Official Publication. Oxford, IN: Association of American Feed Control Officials Inc; 2018.

[4] Kienzle E. Further developments in the prediction of metabolizable energy (ME) in pet food. J Nutr. 2002;132: 1796S–1798S. 10.1093/jn/132.6.1796S 12042532

[5] Atwater WO. Principles of nutrition and nutritive value of food. USDA Farmers’ Bull. 1902;142: 1–48.

[6] Kuhlman G, Laflamme DP, Ballam JM. A simple method for estimating the metabolizable energy content of dry cat foods. Fel Pract. 1993;21: 16–20.

[7] Kendall PT, Burger IH, Smith PM. Methods of estimation of the metabolizable energy content of cat foods. Fel Pract. 1985;15: 38–44.

[8] Kendall PT, Holme DW, Smith PM. Methods of prediction of the digestible energy content of dog foods from gross energy value, proximate analysis and digestive nutrient content. J Sci Food Agric. 1982;33: 823–831.

[9] National Research Council. Nutrient requirements of dogs and cats. Washington, DC: The National Academies Press; 1985.

[10] Kienzle E, Opitz B, Earl KE, Smith PM, Maskell IE, Iben C. The development of an improved method of predicting the energy content in prepared dog and cat food. J Anim Physiol Anim Nutr. 1998;79: 69–79.

[11] Laflamme DP. Determining metabolizable energy content in commercial pet foods. J Anim Physiol Anim Nutr (Berl). 2001;85: 222–230. 11686793 10.1046/j.1439-0396.2001.00330.x

[12] Yamka RM, Mcleod KR, Harmon DL, Freetly HC, Schoenherr WD. The impact of dietary protein source on observed and predicted metabolizable energy of dry extruded dog foods. J Anim Sci. 2007;85: 204–212. 10.2527/jas.2005-336 17179557

[13] Castrillo C, Hervera M, Baucells MD. Methods for predicting the energy value of pet foods. R Bras Zootec. 2009;38S: 1–14.

[14] Kienzle E, Biourge V, Schönmeier A. Prediction of energy digestibility in complete dry foods for dogs and cats by total dietary fiber. J Nutr. 2006;136: 2041S–2044S. 10.1093/jn/136.7.2041S 16772491

[15] European Pet Food Federation (FEDIAF). Nutritional requirements. [Cited 24 April 2019]. Available from: http://www.fediaf.org//prepared-pet-foods/nutritional-requirements.html

[16] Hall JA, Melendez LD, Jewell DE. Using gross energy improves metabolizable energy Predictive equations for pet foods whereas undigested protein and fiber content predict stool quality. PLoS ONE. 2013;8(1): e54405 10.1371/journal.pone.0054405 23342151 PMC3544805

[17] Harrell FE, Lee KL, Mark DB. Multivariable prognostic models: issues in developing models, evaluating assumptions and adequacy, and measuring and reducing errors. Stat Med. 1996;15: 361–387. 10.1002/(SICI)1097-0258(19960229)15:4<361::AID-SIM168>3.0.CO;2-4 8668867

[18] Giancrisotofaro RA, Salmaso L. Model performance analysis and model validation in logistic regression. Statistica. 2003;63: 375–396.

[19] Clinical and Laboratory Standards Institute. Measurement procedure comparison and bias estimation using patient samples; approved guidelines; CLSI document EP09-A3. 3rd ed. Wayne: Clinical Laboratory Standards Institute; 2013.

[20] Martin RF. General Deming regression for estimating systematic bias and its confidence interval in method-comparison studies. Clin Chem. 2000;46: 100–104. 10620577

[21] Bland JM, Altman DG. Statistical methods for assessing agreement between two methods of clinical measurement. Lancet. 1986;327: 307–310. 10.1016/S0140-6736(86)90837-8 2868172

[22] Jensen AL, Kielgaard-Hansen M. Method comparison in the clinical laboratory. Vet Clin Pathol. 2006;35: 276–286. 16967409 10.1111/j.1939-165x.2006.tb00131.x

[23] Naumann C, Bassler R, Seibold R, Cath C. Methodenbuch Band III, Die Chemische Untersuchung von Futtermitteln. Damstadt: VDLUFA-Verlag; 1997; Neudamm: Verlag J. Naumann.

[24] Prosky L, Asp NG, Furda I, DeVries JW, Schweizer TF, Harland BF. Determination of total dietary fiber in foods and food products: collaborative study. J Assoc Off Anal Chem. 1985;68: 677–679. 2993226

[25] Bermingham EN, Thomas DG, Morris PJ, Hawthorne AJ. Energy requirements of adult cats. Br J Nutr. 2010;103: 1083–1090. 10.1017/S000711450999290X 20100376

[26] Bermingham EN, Thomas DG, Cave NJ, Morris PJ, Butterwick RF, German AJ. Energy requirements of adult dogs: a meta-analysis. PLoS ONE. 2014;9(10): e109681 10.1371/journal.pone.0109681 25313818 PMC4196927

[27] German AJ, Holden SL, Mason SL, Bryner C, Bouldoires C, Morris PJ, et al Imprecision when using measuring cups to weigh out extruded dry kibbled food. J Anim Animal Physiol Anim Nutr. 2011;95: 368–373. 10.1111/j.1439-0396.2010.01063.x 21039926

[28] German AJ, Holden SL, Bissot T, Hackett RM, Biourge V. Dietary energy restriction and successful weight loss in obese client-owned dogs. J Vet Int Med. 2007;21: 1174–1180. 10.1111/j.1939-1676.2007.tb01934.x 18196722

