# Correction: Metabolisable energy content in canine and feline foods is best predicted by the NRC2006 equation

**DOI:** 10.1371/journal.pone.0319808

**Published:** 2025-03-19

**Authors:** Juliane Calvez, Mickael Weber, Claude Ecochard, Louise Kleim, John Flanagan, Vincent Biourge, Alexander J. German

In the Feeding trial protocol, proximate analysis and measurement of the energy content of food subsection of Materials and methods, there is an error in the first sentence of the second paragraph. The correct sentence is: The correction factors used for energy lost in the urine were 1.25 and 0.86 Kcal per gram for protein for dogs and cats, respectively.
